# Association of Percentage Body Fat and Metabolic Health in Offspring of Patients with Cardiovascular Diseases

**DOI:** 10.1038/s41598-018-32230-7

**Published:** 2018-09-14

**Authors:** Yuan-Yuei Chen, Wen-Hui Fang, Chung-Ching Wang, Tung-Wei Kao, Yaw-Wen Chang, Hui-Fang Yang, Chen-Jung Wu, Yu-Shan Sun, Wei-Liang Chen

**Affiliations:** 1 Department of Internal Medicine, Tri-Service General Hospital Songshan Branch; and School of Medicine, National Defense Medical Center, Taipei, Taiwan Republic of China; 2 Division of Family Medicine, Department of Family and Community Medicine, Tri-Service General Hospital; and School of Medicine, National Defense Medical Center, Taipei, Taiwan Republic of China; 3 Division of Geriatric Medicine, Department of Family and Community Medicine, Tri-Service General Hospital; and School of Medicine, National Defense Medical Center, Taipei, Taiwan Republic of China; 4 Health Management Center, Department of Family and Community Medicine, Tri-Service General Hospital, National Defense Medical Center, Taipei, Taiwan Republic of China; 5 Graduate Institute of Clinical Medical, College of Medicine, National Taiwan University, Taipei, Taiwan Republic of China; 6 Division of Family Medicine, Department of Community Medicine, Taoyuan Armed Forces General Hospital, Taoyuan, Taiwan Republic of China

## Abstract

Family history was one of the major risk factors for developing adverse health outcomes such as metabolic syndrome (MetS), type 2 diabetes mellitus (DM) and hypertension (HTN). Our aim was to examine the relationship between different family histories and cardiometabolic events, including DM, stroke, myocardial infarction (MI), and HTN. Participants who attended the health examinations at the Tri-Service General Hospital from 2010 to 2016 were enrolled in the study and were categorized into four groups by representing different family history. A multivariable logistic regression model was used for the associations between various family history with the cardiometabolic events. Subjects with family history of DM were divided into quartiles by percentage body fat (PBF) to be analyzed for these adverse outcomes. In the cross-sectional analysis, subjects with family history of DM had significant association with MetS (OR = 1.34 [95%CI: 1.17–1.54]) and DM (OR = 3.03 [95%CI: 2.44–3.76]), and those with family history of HTN were positively associated with HTN (OR = 1.60 [95%CI: 1.41–1.81]). Notably, those with family history of DM in higher PBF quartiles had substantially increased association of cardiometabolic events (MetS: OR = 15.20 [95%CI: 9.87–23.39]; DM: OR = 3.35 [95%CI: 1.91–5.90]; HTN: 2.81 [95%CI: 1.84–4.29]). Individuals with family history of DM were positively associated with MetS and DM, and those with family history of HTN was associated with HTN. Family history assessment was requested especially in obese population for screening adverse health outcomes.

## Introduction

The prevalence of chronic disorders such as metabolic syndrome (MetS), type 2 diabetes mellitus (DM), and hypertension (HTN) had increased in the past decades worldwide^1,2^. A high prevalence of obesity population was noted in Taiwan which was contributed to MetS and DM and became emerging economies and public health problems^3,4^. These cardiometabolic complications were multifactorial and a wide spectrum of different factors including environments, lifestyles, and genetic^5–7^.

Family history, was indisputably one of the major risk factors for chronic diseases like cancer, cardiovascular disease, DM, and was considered as an important genomic tool for preventive medicine and public health recently^8,9^. There were several advantages for family history assessment including inexpensive, greater acceptability, and a reflection of shared genetic and environmental risk factors^10^. Previous studies had reported the impact of family history on the risk of developing adverse health outcomes. In a Korean study, young adults with a family history of DM had an increased risk of DM and MetS^11^. The incidence of DM was increased among individuals with a family history of DM in Caucasians^12^.

Emerging studies reported that increased adiposity was associated with risks of cardiometabolic diseases^13,14^. High percentage body fat (PBF) was correlated with increased risk of DM even having a normal body mass index (BMI)^15^. In the present study, we hypothesized that participants who had family history with high PBF would have more closely association with cardiometabolic events than those with low PBF. Moreover, we examined the associations between combinations of different family histories and cardiometabolic risks. Our main goal was to ascertain the relevance and usefulness of PBF in the relationship between family histories with the cardiometabolic events.

## Results

### Demographic characteristics of the study population

There were 13561 participants with family history of DM, 3775 with family history of MI, 5460 with family history of stroke, and 18399 with family history of HTN. The mean age of each subgroup was 40.68 ± 13.58 (DM), 42.10 ± 13.92 (MI), 42.56 ± 14.18 (stroke), and 42.69 ± 14.37 (HTN). All characteristics of participants in different family history subgroups were shown in Table 1.

**Table 1:** Characteristics of study sample.

Variables	Family history				
	DM	Stroke	MI	HTN	Total
**Continuous Variables, mean (SD)**					
Age (years)	40.68 (13.58)	42.10 (13.92)	42.56 (14.18)	42.79 (14.36)	42.69 (14.37)
LDL-C (mg/dL)	113.61 (31.67)	114.04 (31.23)	114.67 (31.98)	114.08 (31.13)	114.31 (31.46)
Uric acid (mg/dL)	5.53 (1.47)	5.52 (1.49)	5.47 (1.47)	5.56 (1.48)	5.57 (1.48)
Creatinine	0.80 (0.35)	0.80 (0.35)	0.79 (0.31)	0.80 (0.30)	0.81 (0.31)
AST (U/L)	20.48 (13.86)	20.43 (11.42)	20.30 (15.09)	20.72 (16.75)	20.72 (15.72)
Albumin (g/dL)	4.48 (0.29)	4.46 (0.28)	4.46 (0.28)	4.47 (0.29)	4.47 (0.29)
HsCRP (mg/dL)	0.24 (0.49)	0.20 (0.29)	0.23 (0.48)	0.24 (0.54)	0.23 (0.50)
TSH	2.23 (1.63)	2.21 (1.30)	2.26 (1.42)	2.23 (1.48)	2.24 (1.56)
**Category Variables, (%)**					
Gender (male)	5802 (42.8)	1620 (42.9)	2170 (39.7)	8024 (43.6)	11746 (45.0)
Smoking	1644 (28.1)	495 (28.9)	675 (26.4)	2222 (27.3)	3264 (27.9)
Drinking	2443 (48.3)	761 (50.3)	1040 (46.4)	3385 (47.9)	4859 (48.0)

### Association between various family history and cardiometabolic events

In Table 2, a multivariable logistic regression analysis was performed for the association between various family history and MetS, DM, and HTN. The odds ratios (ORs) for MetS in subjects with family history of DM were 1.31 (95%CI: 1.17–1.47), 1.35 (95%CI: 1.17–1.55) and 1.34 (95%CI: 1.17–1.54) in each adjusted model. However, other family history was not significantly associated with MetS. The association between DM and family history of DM were remained significant after adjustment for various covariables with ORs of 2.46 (95%CI: 2.02–3.01), 3.05 (95%CI: 2.46–3.78), and 3.03 (95%CI: 2.44–3.76) in each model. In the outcome of HTN, only those with family history of HTN had significant difference with ORs of 1.60 (95%CI: 1.42–1.79), 1.60 (95%CI: 1.42–1.81), and 1.60 (95%CI: 1.41–1.81) after multivariable adjustment.

**Table 2:** Association between family history and the presence of different health outcomes.

Variables of family history	Model^a^ 1 OR (95% CI)	*P* Value	Model^a^ 2 OR (95% CI)	*P* Value	Model^a^ 3 OR (95% CI)	*P* Value
***MetS***						
DM	1.310 (1.165–1.473)	<0.001	1.346 (1.172–1.545)	<0.001	1.338 (1.165–1.537)	<0.001
Stroke	1.043 (0.859–1.266)	0.672	0.987 (0.785–1.240)	0.909	0.978 (0.777–1.231)	0.851
MI	1.033 (0.881–1.210)	0.692	0.965 (0.800–1.164)	0.710	0.959 (0.794–1.158)	0.663
HTN	1.273 (1.141–1.420)	<0.001	1.093 (0.961–1.243)	0.175	1.089 (0.957–1.240)	0.194
***DM***						
DM	2.463 (2.016–3.008)	<0.001	3.049 (2.456–3.784)	<0.001	3.031 (2.441–3.763)	<0.001
Stroke	0.876 (0.601–1.276)	0.489	0.924 (0.625–1.365)	0.690	0.933 (0.631–1.379)	0.729
MI	1.059 (0.795–1.411)	0.693	1.082 (0.801–1.463)	0.606	1.085 (0.802–1.466)	0.597
HTN	1.000 (0.817–1.224)	0.999	0.988 (0.798–1.223)	0.915	0.994 (0.803–1.231)	0.958
***HTN***						
DM	0.887 (0.780–1.009)	0.068	0.899 (0.783–1.031)	0.127	0.900 (0.0.785–1.033)	0.135
Stroke	0.966 (0.785–1.190)	0.747	0.963 (0.772–1.201)	0.738	0.959 (0.769–1.197)	0.712
MI	1.013 (0.857–1.198)	0.877	1.027 (0.858–1.228)	0.774	1.026 (0.857–1.228)	0.781
HTN	1.596 (1.423–1.790)	<0.001	1.602 (1.416–1.813)	<0.001	1.599 (1.412–1.810)	<0.001

^a^Adjusted covariates.

Model 1 = age + gender + BMI.

Model 2 = Model 1 + proteinuria, serum total cholesterol, uric acid, creatinine, AST, albumin, hsCRP.

Model 3 = Model 2 + history of smoking, drinking.

### Subjects with family history of DM in PBF quartiles and cardiometabolic events

Participants with family history of DM were divided into quartiles by PBF. Associations between these people and cardiometabolic events were analyzed in Table 3. The ORs for MetS in subjects with different PBF quartiles after full adjustment were Q2: 2.82, Q3: 5.08 and Q4: 15.20 (95%CI: 2.03–3.91, 3.53–7.33, 9.87–23.39). Only subjects in the highest quartile of PBF were significantly associated with DM with ORs of 3.35 (95%CI: 1.91–5.90) after fully adjusting. The ORs for HTN in subjects with different PBF quartiles after fully adjusting were Q1: 1.50, Q2: 1.81, and Q3: 2.81 (95%CI: 1.08–2.09, 1.26–2.61, 1.84–4.29).

**Table 3:** Association between family history of DM and the presence of different health outcomes in PBF quartiles.

Variables	PBF in quartiles	Model^a^ 1 OR (95% CI)	*P* Value	Model^a^ 2 OR (95% CI)	*P* Value	Model^a^ 3 OR (95% CI)	*P* Value
Family history of DM (+)	***MetS***						
	Q2 v.s. Q1	1.901 (1.406–2.571)	<0.001	2.696 (1.946–3.734)	<0.001	2.816 (2.026–3.914)	<0.001
	Q3 v.s. Q1	2.346 (1.731–3.179)	<0.001	4.803 (3.349–6.890)	<0.001	5.084 (3.527–7.330)	<0.001
	Q4 v.s. Q1	4.702 (3.455–6.398)	<0.001	14.260 (9.312–21.838)	<0.001	15.198 (9.874–23.394)	<0.001
Family history of DM (+)	***DM***						
	Q2 v.s. Q1	0.976 (0.646–1.474)	0.907	1.284 (0.823–2.004)	0.271	1.307 (0.836–2.043)	0.241
	Q3 v.s. Q1	0.785 (0.502–1.228)	0.289	1.067 (0.642–1.775)	0.802	1.094 (0.655–1.828)	0.731
	Q4 v.s. Q1	1.412 (0.937–2.129)	0.099	3.247 (1.853–5.691)	<0.001	3.352 (1.905–5.898)	<0.001
Family history of DM (+)	***HTN***						
	Q2 v.s. Q1	1.199 (0.876–1.641)	0.256	1.529 (1.098–2.130)	0.012	1.499 (1.075–2.090)	0.017
	Q3 v.s. Q1	1.211 (0.878–1.671)	0.243	1.863 (1.297–2.675)	<0.001	1.812 (1.260–2.607)	<0.001
	Q4 v.s. Q1	1.509 (1.090–2.090)	0.013	2.873 (1.884–4.381)	<0.001	2.812 (1.843–4.291)	<0.001

^a^Adjusted covariates:

Model 1 = age + gender + BMI.

Model 2 = Model 1 + proteinuria, serum total cholesterol, uric acid, creatinine, AST, albumin, hsCRP.

Model 3 = Model 2 + history of smoking, drinking.

### Association between various combinations of family history and DM

In Table 4, we categorized participants into various combinations of family history (DM, stroke, HTN, and MI). Associations between various combinations of family history and DM were analyzed by a univariate and a multivariate logistic regression model, respectively. Combinations which composed of family history of DM were significantly associated with DM in univariate model: C2 (DM): ORs = 3.461 (95%CI: 2.614–4.584), C6 (DM + MI): ORs = 2.636 (95%CI: 1.239–5.608), C10 (DM + HTN): ORs = 1.963 (95%CI: 1.419–2.717), and C14 (DM + HTN + MI): ORs = 2.944 (95%CI: 1.855–4.673). After fully adjusting for pertinent covariates, combinations of family history were associated with DM in C2 (DM): ORs = 4.366 (95%CI: 3.231–5.901), C6 (DM + MI): ORs = 2.782 (95%CI: 1.253–6.174), C10 (DM + HTN): ORs = 2.445 (95%CI: 1.735–3.445), C12 (DM + stroke + HTN): ORs = 2.836 (95%CI: 1.361–5.908), and C14 (DM + HTN + MI): ORs = 4.748 (95%CI: 2.910–7.745). However, subjects with family history combinations of (DM + stroke), (DM + stroke + MI), and (DM, stroke, HTN, MI) were not significantly associated with DM.

**Table 4:** Association between various combinations of family history and DM.

Combination	DM	Stroke	HTN	MI	Univariate analysis^a^ OR (95% CI)	*P* Value	Multivariate analysis^b^ OR (95% CI)	*P* Value
1 (N = 41885)	—	—	—	—	Reference	—	Reference	—
2 (N = 5084)	+	—	—	—	3.461 (2.614–4.584)	<0.001	4.366 (3.231–5.901)	<0.001
3 (N = 767)	—	+	—	—	0.576 (0.181–1.838)	0.351	0.489 (0.149–1.609)	0.239
4 (N = 287)	+	+	—	—	1.656 (0.505–5.426)	0.405	2.952 (0.876–9.946)	0.081
5 (N = 1320)	—	—	—	+	1.104 (0.587–2.076)	0.759	1.267 (0.659–2.435)	0.477
6 (N = 428)	+	—	—	+	2.636 (1.239–5.608)	0.012	2.782 (1.253–6.174)	0.012
7 (N = 123)	—	+	—	+	3.486 (1.108–11.937)	0.047	5.444 (1.545–19.182)	0.008
8 (N = 68)	+	+		+	2.453 (0.308–19.515)	0.396	3.986 (0.471–33.756)	0.205
9 (N = 8267)	—	—	+	—	1.102 (0.801–1.516)	0.550	1.200 (0.862–1.671)	0.279
10 (N = 5366)	+	—	+	—	1.963 (1.419–2.717)	<0.001	2.445 (1.735–3.445)	<0.001
11 (N = 939)	—	+	+	—	1.472 (0.732–2.959)	0.278	1.541 (0.745–3.186)	0.244
12 (N = 879)	+	+	+	—	1.759 (0.871–3.549)	0.115	2.836 (1.361–5.908)	0.005
13 (N = 1437)	—	—	+	+	0.721 (0.349–1.491)	0.378	0.682 (0.317–1.465)	0.327
14 (N = 1477)	+	—	+	+	2.944 (1.855–4.673)	<0.001	4.748 (2.910–7.745)	<0.001
15 (N = 304)	—	+	+	+	1.051 (0.252–4.393)	0.945	1.353 (0.315–5.807)	0.684
16 (N = 595)	+	+	+	+	0.330 (0.045–2.393)	0.273	0.521 (0.070–3.854)	0.523

^a^Adjusted covariates: unadjusted.

^b^Adjusted covariates: age + gender + BMI + proteinuria, serum total cholesterol, uric acid, creatinine, AST, albumin, hsCRP + history of smoking, drinking.

### Association between different cut-off values of PBF and the presence of MetS, DM, and HTN in subjects with family history of DM

To predict the cardiometabolic risks in subjects with family history of DM, we assessed the cut-off values of PBF for the presence of MetS, DM, and HTN by using receiver operating characteristic (ROC) curve analysis. The cut-off values of PBF for MetS, DM, and HTN were 29.05, 32.65, and 29.75, respectively. After fully adjusting for covariates, family history of DM was significantly associated with MetS, DM, and HTN with ORs of 4.351 (95%CI: 3.290–5.753), 2.033 (95%CI: 1.355–3.048), and 1.763 (95%CI: 1.327–2.343), respectively (Table 5).

**Table 5:** Association between different cut-off values of PBF and the presence of MetS, DM, and HTN in subjects with family history of DM.

Variables	PBF Cut-off values	Model^a^ 1 OR (95% CI)	*P* Value	Model^a^ 2 OR (95% CI)	*P* Value	Model^a^ 3 OR (95% CI)	*P* Value
		*MetS*					
Family history of DM (+)	PBF = 29.05	2.565 (2.067–3.183)	<0.001	4.232 (3.208–5.582)	<0.001	4.351 (3.290–5.753)	<0.001
		***DM***					
	PBF = 32.65	1.254 (0.916–1.717)	0.157	1.995 (1.335–2.982)	<0.001	2.033 (1.355–3.048)	<0.001
		***HTN***					
	PBF = 29.75	1.245 (0.989–1.567)	0.062	1.793 (1.351–2.380)	<0.001	1.763 (1.327–2.343)	<0.001

^a^Adjusted covariates:

Model 1 = age + gender + BMI.

Model 2 = Model 1 + proteinuria, serum total cholesterol, uric acid, creatinine, AST, albumin, hsCRP.

Model 3 = Model 2 + history of smoking, drinking.

## Discussion

In the current study, we observed that those with family history of DM had significant association with MetS and DM, and those with family history of HTN were positively associated with HTN. Subjects with family history of DM in higher PBF quartiles were associated with these cardiometabolic events. In addition, family history combinations which contained family history of DM had significant association with the presence of DM. Subjects who had family history of DM with different cut-off values of PBF could predict the risks of MetS, DM, and HTN. Our finding was the first to examine the association between various family history and cardiometabolic events in Taiwanese general population.

Previous researches had reported the important role of not only family history of HTN but also family history of DM in predicting the risks of different adverse health outcomes. In a long-term prospective study, both paternal and maternal hypertension were significantly associated with higher blood pressure and with the development of HTN over the adult life course^16^. Ranasinghe *et al*. had demonstrated that the prevalence of HTN was higher in individuals with family history of HTN^17^. The family history of DM was suggested as a useful tool to evaluate the risks of cardiometabolic disorders such as MetS, DM and cardiovascular diseases^6,18,19^. In a Korean study composed of a young adult population, those with family history of DM had increased risks of MetS and DM^11^. These results were consistent with our findings that the prevalence of MetS and DM was greater in subjects with family history of DM, and those with family history of HTN were correlated with the presence of HTN.

Several studies had addressed the synergistic effect of both positive family history of DM and obesity on the risk of developing DM. Hilding *et al*. demonstrated that exposure to both family history of DM and BMI showed the strongest effect on the risk of developing pre-diabetes and DM and conveyed a higher risk than either alone^20^. In an American study composed of a Indians population, family history of DM was correlated with higher incidence of DM with increased BMI than in those without family history of DM^21^. Obesity had been indicated to be associated with family history of DM and might be substantial part of the association between family history and the risk of DM^22,23^. In a Japanese cohort study, a family history of DM was associated with the incident risk of DM, and this association was independent of interactions with obesity and lifestyle factors^24^. Generalized adiposity reflected by BMI contributed to the association between family history of DM and the risk of developing DM^25^. A similar finding observed by Rice *et al*. represented that total body fat shared common familial determinants^26^. Consistent with our findings, combined family history of DM and higher PBF had increased risk for predicting MetS, DM and HTN than those in lower PBF quartiles. It was tempting to speculate that existing family history increased cardiometabolic risks and ensuing higher PBF could result in metabolic derangement and endothelial dysfunction that harbored a predisposing milieu for cardiometabolic diseases.

Several limitations were noted among the current study. First, a causal inference was not suitable in the study due to a cross-sectional design. A longitudinal analysis was necessary for further researches to examine the association between family history and the risks of adverse health outcomes. Second, the results might be influenced by recall bias because self-reported data was performed in the study to categorize family history which could result in some misclassification. Thus, we validated the family history collected by the questionnaire to eliminate inaccurate information. Third, the study sample was obtained from adult population in Taiwan. The limited ethnicity distribution of participants might not present the effect of family history on adverse health outcomes in terms of racial differences. Next, we only recruited study sample from a single hospital, which could limit generalization to the Taiwanese general population. Last, detail family histories in father and mother and familial risk classification were not recorded. It cannot be provided because our dataset included no information on detail family histories in first-degree relative or second degree relative. Familial risk classifications were not categorized in our analyzed models.

## Conclusion

By assessing the Taiwanese adult population, the current study highlighted that family history of DM was significantly associated with cardiometabolic events. Distinctly, body fat accumulation obviously contributed to increased risks of MetS, DM and HTN, especially in offspring of patients with DM. Prevention strategies of cardiovascular diseases would benefit from giving more attention to lower body fat percentage in individuals with family history. The potential role of reducing PBF in the prevention of cardiometabolic diseases warranted more longitudinal surveys to explore the clinical applications.

## Methods

### Subjects Enrollment

Subjects enrolled in the study were derived from the health examinations at TSGH, from 2010 to 2016. Participants aged 20 years old and older attended comprehensive examinations including laboratory data, body composition and detailed self-reported questionnaires. All protocols in this retrospective study were approved by the Institutional Review Board (IRB) of TSGH. The IRB waived the need to obtain individual informed consent because the data were analyzed anonymously. All methods were performed in accordance with the relevant guidelines and regulations of TSGH.

### Study Design

**Figure 1:**
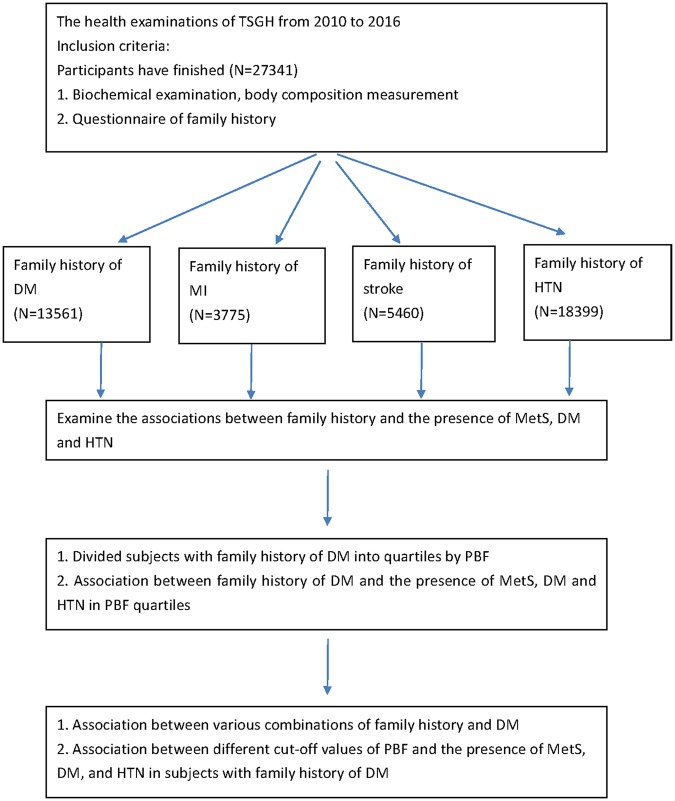
Flow chart of our study design.

According to the flowchart shown in Fig. 1, participants who attended the health examinations at the TSGH from 2010 to 2016 and finished biochemical examination, body composition measurement, and questionnaire of family history were included (N = 27341). Eligible participants were divided into subgroups based on various family history including type 2 diabetes mellitus (DM) (N = 13561), stroke (N = 3775), myocardial infarction (MI) (N = 5460), and hypertension (HTN) (N = 18399). In the next step, a multivariable logistic regression model was performed for the association among different family histories and the presences of MetS, DM and HTN.

### Family History Assessment

Family history was obtained from participants who attended the health examinations at TSGH by using a detailed self-reported questionnaire. A question “What family history do you have” was used for assessing various kinds of family history.

### Definition of MetS

Several organizations had various criteria for diagnosing MetS. The World Health Organization (WHO) first reported its definition in 1998^27^. The National Cholesterol Education Program (NCEP) Adult Treatment Panel III (ATP III) presented another definition in 2001 and updated in 2005^28^. Subsequently, the International Diabetes Foundation (IDF) demonstrated an new criteria in 2005^29^. In the current study, we adopted the nationwide standard published by the Taiwan Health Promotion Administration of the Ministry of Health and Welfare in 2007^30^. A subject with at least 3 of the abnormal components was diagnosed of MetS: (1) blood pressure ≥130/85 mmHg; (2) waist circumference (WC) >90 cm for males and >80 cm for females; (3) fasting plasma glucose ≥100 mg/dL; (4) HDL-C<40 mg/dL for males and <50 mg/dL for females; and (5) triglyceride ≥150 mg/dL.

### Definition of DM

Subjects with type 2 DM was diagnosed by the American Diabetes Association criteria: (1) fasting plasma glucose ≥126 mg/dL; (2) 2 hours plasma glucose ≥200 mg/dL during oral glucose tolerance test 75 g; (3) hemoglobin A1c tes t ≥ 6.5%; and (4) random plasma glucose ≥200 mg/dL^31^.

### Definition of HTN

The definition of HTN in the current study was adopted by the guidelines which blood pressure ≥ 140/90 mmHg or subjects taking antihypertensive agents^32^.

### Measurement of Body Composition

PBF was measured by bioelectrical impedance analysis (BIA) (InBody720, Biospace, Inc., Cerritos, CA, USA), an useful method for assessing body composition^33^. The procedure of BIA was simple and noninvasive, and the results were reproducible and rapidly obtained.

### Assessments of Covariates

These pertinent characteristics included demographic factors (age, gender), biochemistry data (body mass index (BMI), proteinuria, serum total cholesterol, uric acid, creatinine, aspartate aminotransferase (AST), albumin, highly sensitive C-reactive protein (hsCRP)), and personal history (cigarette smoking, alcoholic consumption). A self-reported questionnaire was used to obtain age, gender and personal history. BMI was calculated by a formula that the weight divided by the square of the height (kg/m^2^) of a participant. Subjects were asked to fast at least 8 hours before health examinations for collecting blood samples. Biochemistry data was analyzed by different standard measurements. Total cholesterol was analyzed by an enzymatic colorimetric method (Roche Diagnostics, Indianapolis, IN, USA). The latex-enhanced nephelometry was used to detect hsCRP. Serum uric acid was measured by the Hitachi 737 automated multichannel chemistry analyzer (Boehringer Mannheim Diagnostics, Indianapolis, IN, USA).

### Statistical Analysis

We classified subjects into different family history and compared the distribution of characteristics and covariates across subgroups by using ANOVA for continuous variables and the chi-squared test for categoric variables. Statistical significance was defined as a two-sided *p*-value of ≤0.05. Multivariable models were adjusted as follows: Model 1 included age, gender and BMI. Model 2 included Model 1 plus proteinuria, serum total cholesterol, uric acid, creatinine, AST, albumin and hsCRP. Model 3 included Model 2 plus history of cigarette smoking and alcoholic consumption. A logistic regression model was investigated for the association between family history and the risk of developing MetS, DM and HTN. Analyses in the current study were conducted by Statistical Package for the Social Sciences, version18.0 (SPSS Inc., Chicago, IL, USA) for Windows.
